# Utilizing interdigital and supershape geometries for the design of frequency selective surfaces with high angular and polarization stabilities

**DOI:** 10.1038/s41598-022-10960-z

**Published:** 2022-04-29

**Authors:** Amir Khajevandi, Homayoon Oraizi

**Affiliations:** School of Electrical Engineering, Iran University of Science and Technology, Tehran, 1684613114 Iran

**Keywords:** Engineering, Electrical and electronic engineering

## Abstract

In this research article, the superformula is used to design the geometry of a frequency selective surface (FSS) unit cell, which resembles the shapes found in nature. The designed shape of unit cell is like petals, which may take different form by varying the values of six parameters. The proposed FSS unit cell has both angular and polarization stabilities of incident wave. For the miniaturization of FSS and decrease of resonance frequency, interdigital capacitances (IDCs) are devised in the FSS structure, which do not deteriorate angular and polarization stabilities. The dimensions of the unit cell are 10 mm × 10 mm and the resonance frequency is specified as 3.5 GHz. An equivalent circuit is derived for the unit cell to evaluate its frequency responses. Its performance as the transmission coefficient is obtained by the equivalent circuit and full-wave simulation. The effects of variations of the geometrical dimensions of the FSS unit cell on its performance are studied. A prototype model of proposed FSS is fabricated and measured. The performance of its equivalent circuit, full-wave computer simulation results and measured data are compared and are shown to be in good agreement.

## Introduction

Frequency selective surfaces (FSSs) are periodic structures commonly designed by metallic elements on a dielectric substrate. Indeed, they are spatial filters possessing spectral characteristics of low pass, high pass, band pass, band reject or all pass for single or multi band performances^1^. Various responses are realized by different unit cell element shapes and geometries, such as ring, square patch, fractal slots or patches. Different structures and geometrical configurations have been proposed for FSSs. They often display selectivity on the angle and polarization of the incident wave. However, for the application of FSS as a filter with particular characteristics, several specifications are desirable, such as angular stability, polarization stability, broad banding and miniaturization^2–4^.

FSSs can be used in many applications, such as antennas, reflectors, absorbers, radomes, shielding applications, metamaterials, isolation of unwanted and harmful radiations in hospitals, schools and domestic environments^5–11^ in various frequency ranges. Due to their extensive applications at various frequencies, their investigations have been popular in microwave and optical problems for decades, such as the communication capabilities of satellite platforms. In the FSS design, an important consideration is the unit cell miniaturization, due to the practical space limitations. However, an FSS structure should ideally be designed with an infinite number of unit cells. Consequently, it is necessary to obtain the response of an infinitely large FSS by a finite number of unit cells. In the process of FSS miniaturization, the angular stability should also be realized^12^, but at high angles of incidence, the resonance frequencies usually change, due to the unit cell geometry. One of the methods implemented for the broad banding of FSSs is the application of fractal geometries for the unit cell, which also brings about miniaturization^13,14^. Generally, for the design of FSSs with particular specifications, such as band-stop and bandwidth responses, various configurations such as planar and ring types have been used.

The employment of superformula for the design of FSS unit-cells is unprecedented in the related literature and is quite original. Its application for the design FSSs provides for their superior performance. In the available literature, the fractal geometries and other configurations have been used to design FSS unit-cells which lead to miniaturization and concurrently, the angular stabilities were also achieved. The design of FSSs by supershapes provide several properties simultaneously, such as miniaturization and angular stability. The application of superformulas for the design of FSSs leads to novel and interesting geometrical configurations which are quite distinct from the simple common shapes and also fractal geometries.

In this paper, a stop-band FSS with excellent performance is proposed. The proposed FSS configuration is derived by the superformula and its structure is built on a substrate with one metallized surface. Its shape resembles a petal. It has a very good angular stability for the incident angle of 0° to 80°. The parameters of the superformula may be varied to obtain the desired shape. By the use of interdigital capacitors (IDC) on the FSS structure, its resonance frequency may be reduced by about 1 GHz. Such IDCs greatly affect the reduction of resonance frequency, more specifically the IDCs and resonance frequency will change by varying the spacings and widths of metallic traces. This electrical behavior will be interpreted by devising appropriate equivalent circuit. In order for the FSS to possess polarization stability, it should be symmetrical with respect to the *x* and *y* axes in the plane. The proposed FSS element shape should possess such properties. The dimensions of the unit cell will be taken as 10 mm × 10 mm and the resonance frequency will be set as 3.5 GHz.

## Design of FSS element shape by the superformula

**Figure 1:**
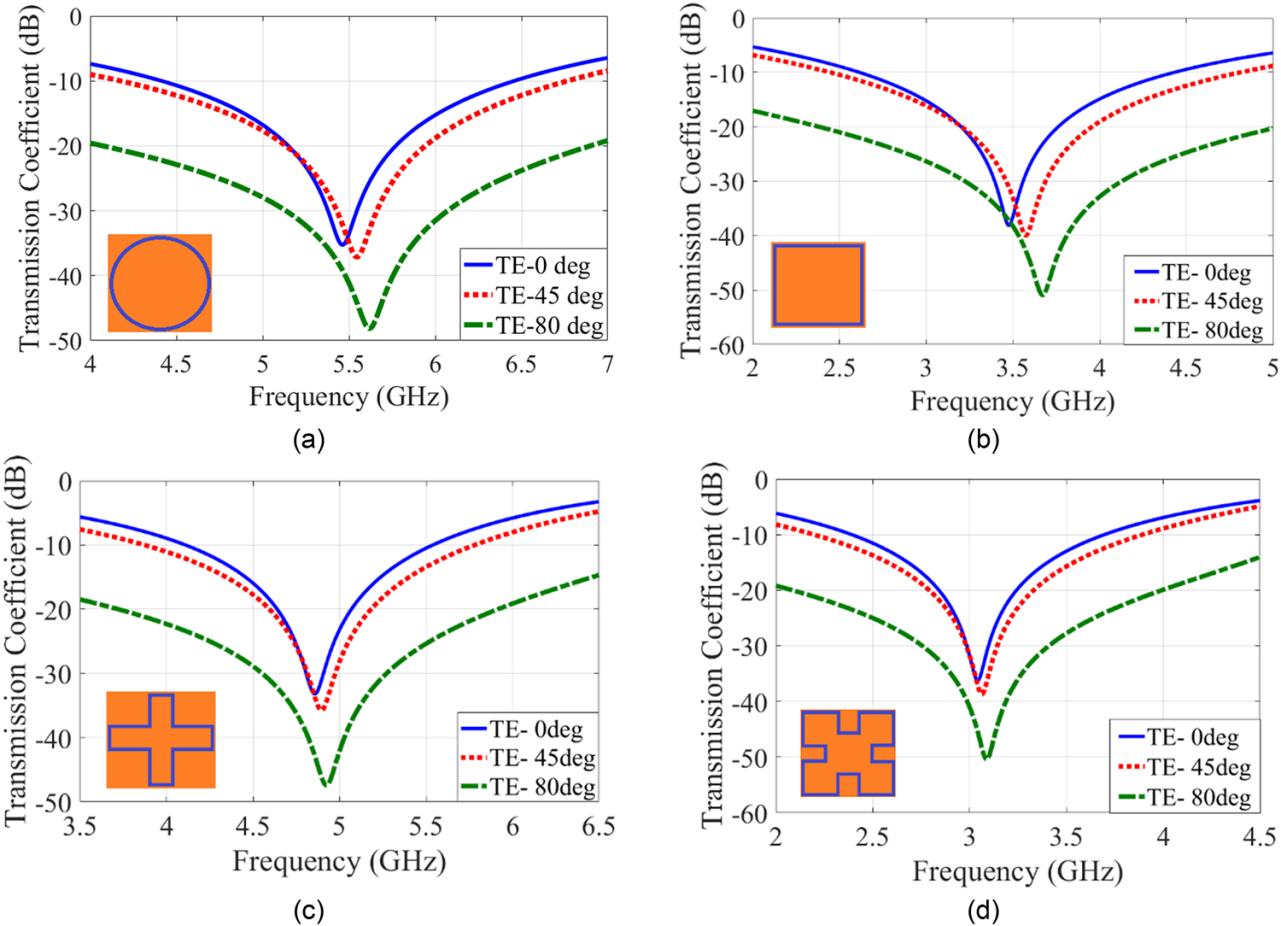
Transmission Coefficient for: (**a**) circle, (**b**) square, (**c**) four legged loaded, (**d**) Minkowski fractal.

Figure 1 shows the transmission coefficient of various closed-loop FSSs using similar structure and technology (namely single-layer and single-sided) for better comparison with the square, circular and Minkowski fractals with four loaded-legs having sides equal to 10 mm for several angles of incidence for the TE mode. Observe that the resonance frequency varies with the incidence angle. Observe also that although the Minkowski unit cell has the lowest resonance frequency and indeed has the best angular-stability compared to the other shapes due to miniaturization, but its resonance frequency changes at the higher incidence angles.

**Figure 2:**
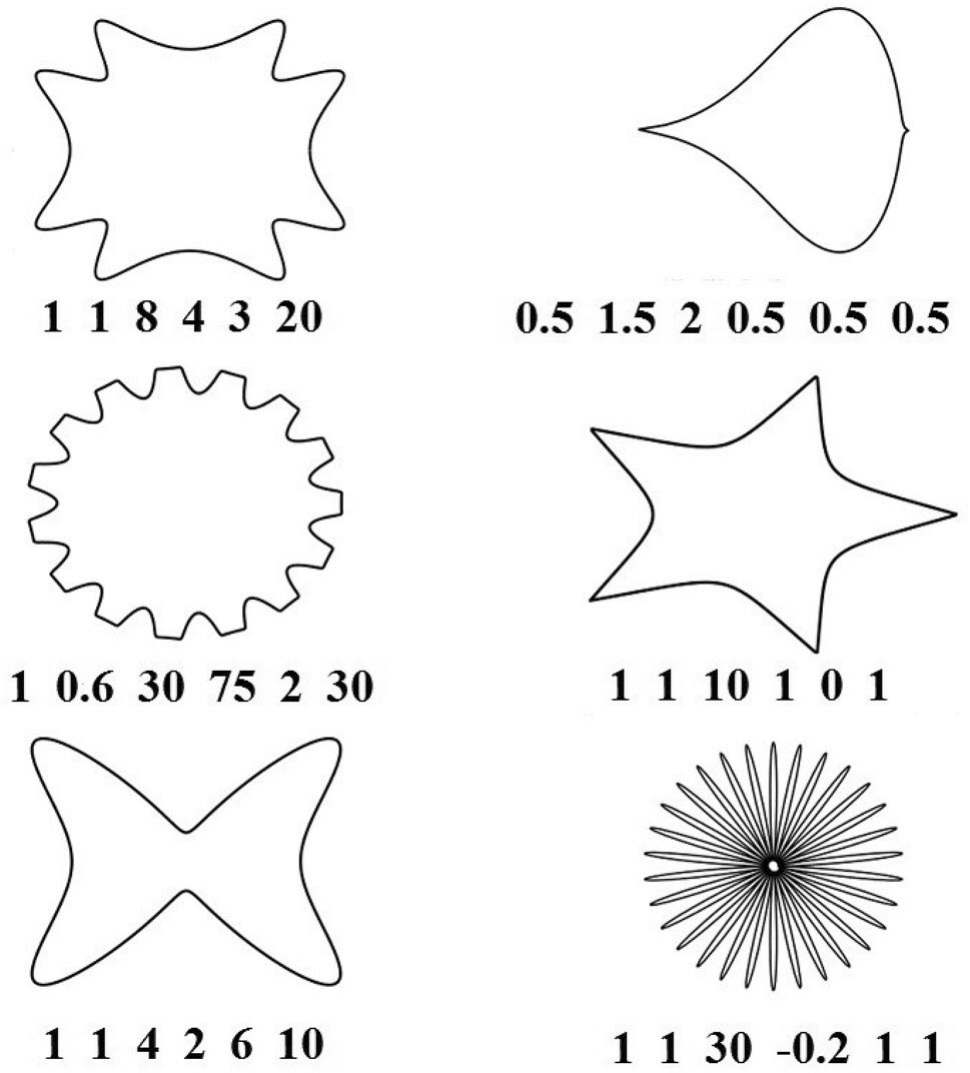
Sample of shapes produced by superformula. Their corresponding a, b, m, n1, n2, n3 are shown under each shape respectively.

The common geometries such as square, rectangle, circle and polygon have already been used and their performances have been extensively investigated for the FSS unit cell. Fractal geometries were originally inspired by figures observable in nature and have been used for the design of unit cell. However, nature-inspired shapes may provide opportunities for the designer to realize desirable characteristics. In^15^ Johan Gielis presented a new geometrical approach for modeling and understanding various abstract, natural, and man-made shapes. Starting from the concept of the circle, he showed that a large variety of shapes can be described by a single and simple geometrical equation, namely the so-called superformula. The variations of its parameters permit the generation of various natural polygons. The mathematics behind the superformula are easily understood and given the wide range of applications, both in technology and science like biological organisms and geometric morphometric, indeed the superformula has the potential to transform the perception of symmetry and shape in a profound manner, because of increasing the degrees of freedom. Recently supershapes have been used in the design of antennas^16–18^ and FSSs^19,20^. Indeed, observable figures in nature have served as inspiration for the selection of shapes for radiating elements. The Fourier series may be used for the generation of natural shapes, but many terms are required which lead to high computation burden. On the contrary, the superformula may generate many natural shapes by the specification of only six parameters. A superformula allowing to generate naturally occurring geometrical shapes is introduced in Fig. 2.

The superformula in the polar coordinates generating the supershapes is^15^:

1 $$r\left(\theta \right)={\left({\left|\frac{\mathrm{cos}\left(\frac{m\theta }{4}\right)}{a}\right|}^{n2}+{\left|\frac{\mathrm{sin}\left(\frac{m\theta }{4}\right)}{b}\right|}^{n3}\right)}^{-\frac{1}{n1}}$$

It depends on six parameters, *m*, *n*_1_, *n*_2_, *n*_3_, *a* and *b*. It has been devised to generate the geometrical shapes and figures appearing in nature by varying the values of these six parameters. The parameter *m* determines the number of lobes, say in the petal. The other parameters determine the degrees of curvatures and clefts of the figures and shapes of FSS unit cells. The shape of figure obtained for FSS by the superformula for the selected values of six parameters determines its frequency response.

**Figure 3:**
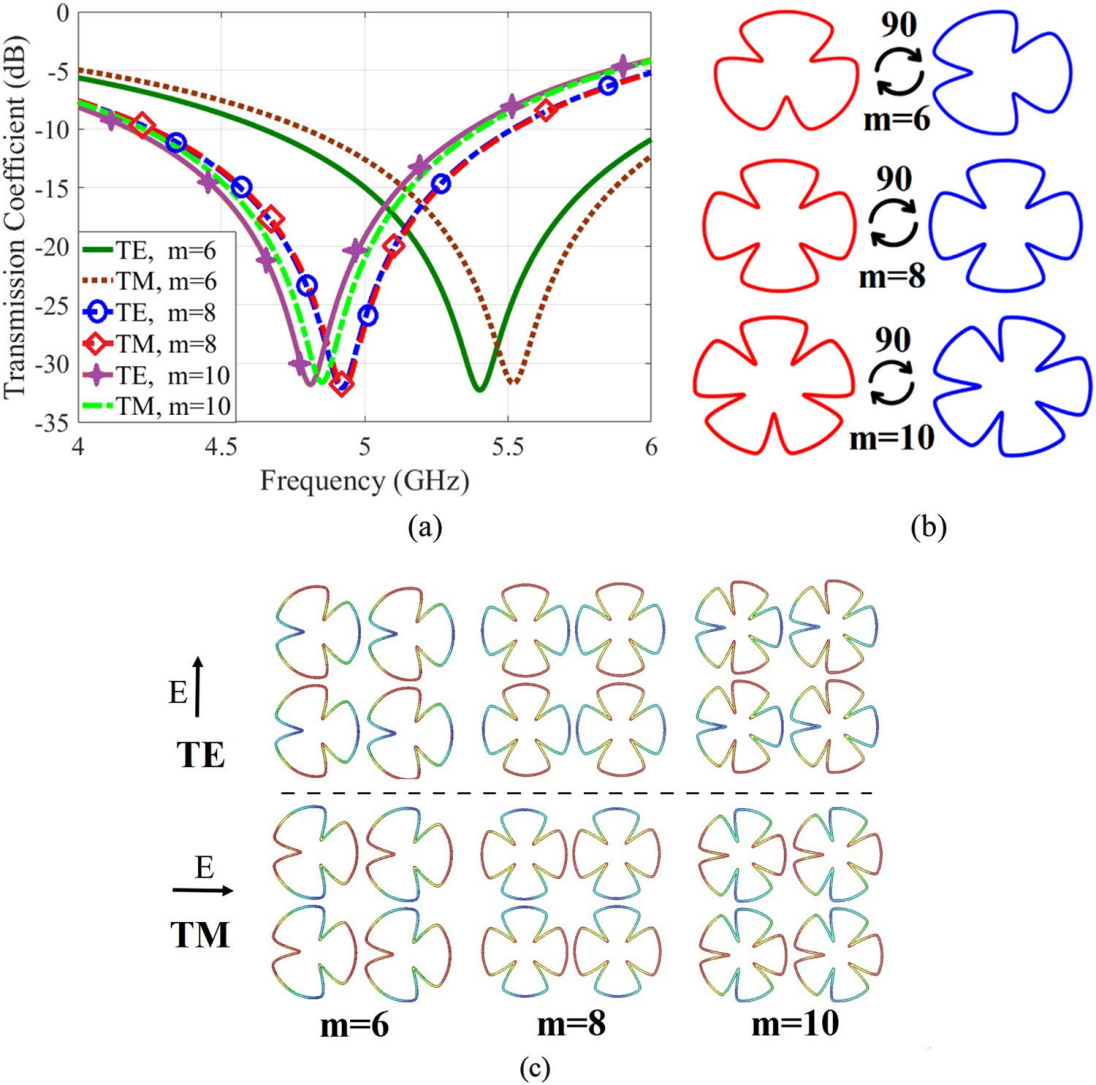
(**a**) Supershape transmission coefficients at different values of *m*, (**b**) 90° rotation of supershape, (**c**) electrical field distribution for different values of m for unit cell for TE and TM modes.

The transmission coefficients of several supershapes (different values of *m*) are shown in Fig. 3. They are all circumscribed in a circle of diameter 10 mm. The related parameters of superformula are *n*_1_ = 0*.*75, *n*_2_ = 0, *n*_3_ = 30, *a* = 1 and *b* = 1. The FR-4 substrate is used having dielectric constant *ε*_*r*_ = 4*.*3, height *h* = 1*.*6 mm and loss tangent tan *δ* = 0*.*02.

Observe that by increasing the value of ‘m’, the resonance frequency decreases. Also for ‘m = 8’, the resonance frequencies of both TE and TM modes are equal (Fig. 3a). Also for such supershape structures, that one with the lowest value of ‘m = 8’ is polarization independent. This property repeats for multiple values of 8. The main reason of this property is the exclusive geometry of this type of supershape. Indeed when ‘m’ is equal to a multiple of 8, the supershape geometrical configuration is invariant and fixed with respect to its rotation by 90° on the unit cell plane. Therefore, for TE and TM modes the unit cell behaves similarly as depicted in Fig. 3b.

The polarization stability may be explained by the proposed equivalent circuit of unit cell. The resonance frequency depends on the geometry of unit cell, which is determined by the equivalent circuit composed of interconnection of inductors and capacitors components depending on the particular incident mode. Figure 3c shows the electrical field distribution on the unit cell for the TE and TM modes. Observe that the electrical field distributions do not possess identical configurations in the tracks and edges of unit cell for m = 6 and m = 10. Consequently, the arrangements of capacitor and inductor components and their values for TE and TM modes are different. However, for m = 8 the electrical field distribution and equivalent circuits and component values are identical for TE and TM modes. In fact, according to Eqs. (1) and (2) the values of inductor and capacitor components depend on the incident angles, the effect of lengths of paths of incident waves, the spacings of tracks and widths of tracks. Since the shapes of unit-cells are curved, their responses strongly depend on the incidence angles, which determine the electrical field distributions on the unit-cells that explained in equivalent circuit model section.

**Figure 4:**
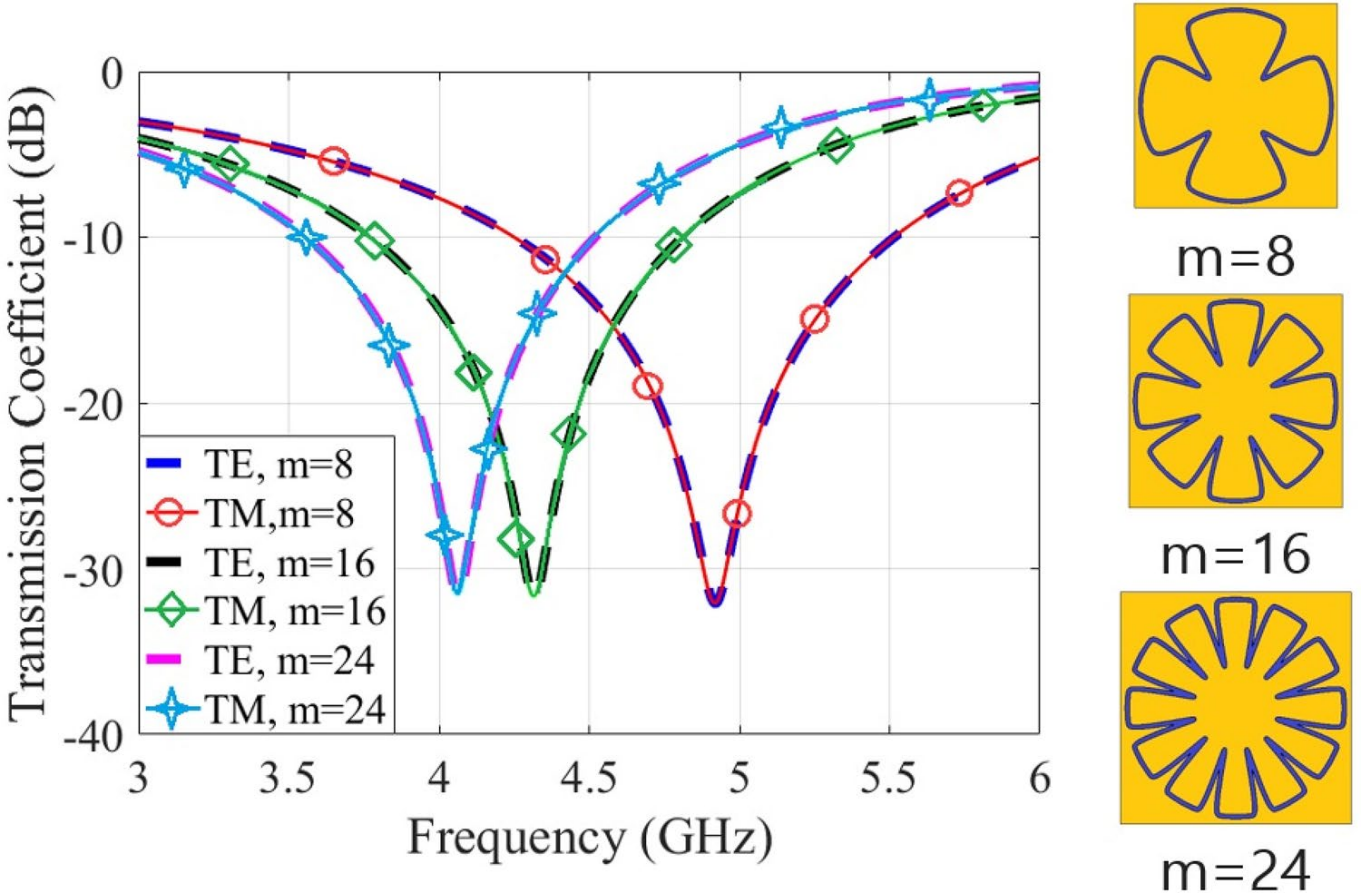
Supershapes transmission coefficients for multiples of m = 8 values.

Figure 4 shows the transmission coefficients of unit-cells for multiple values of ‘m = 8’. The variations of resonances for the values of m = 16 to m = 24 are small. Besides, the fabrication of unit-cells for the higher values of m becomes harder. Therefore, the value of m = 16 is adopted for the proposed FSS geometry.

**Figure 5:**
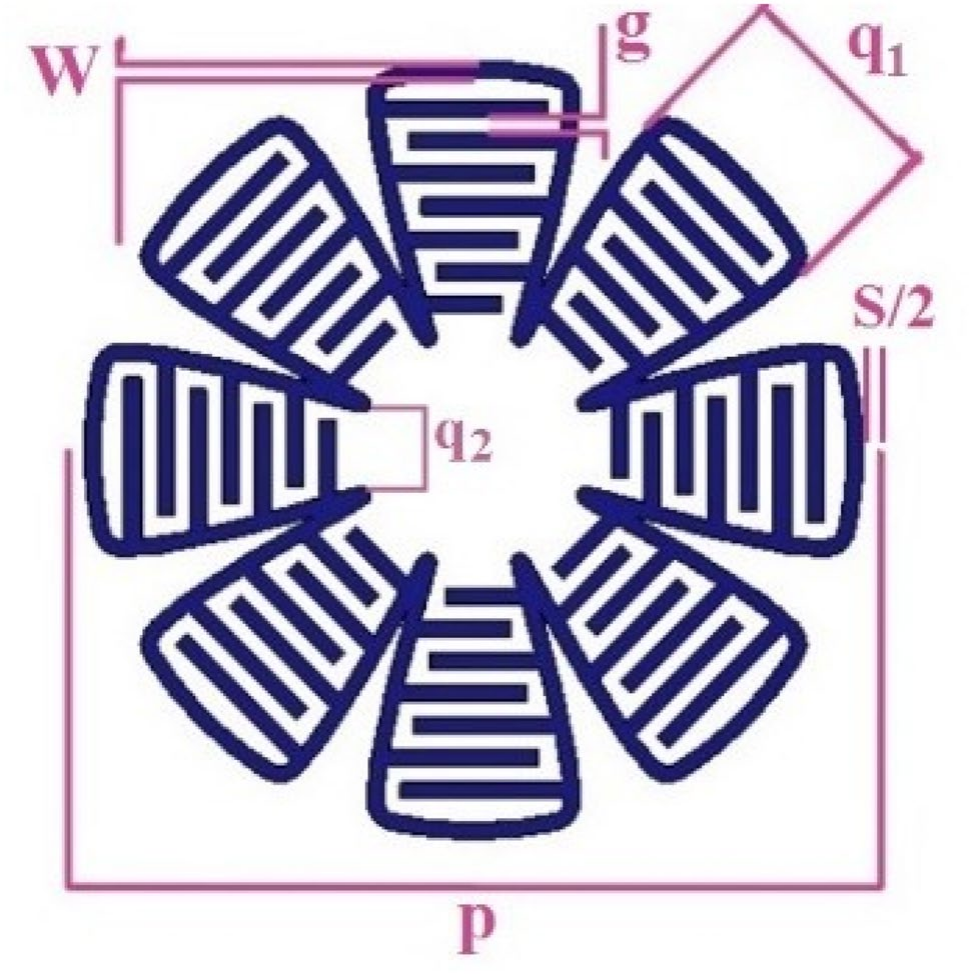
Proposed FSS unit cell.

**Figure 6:**
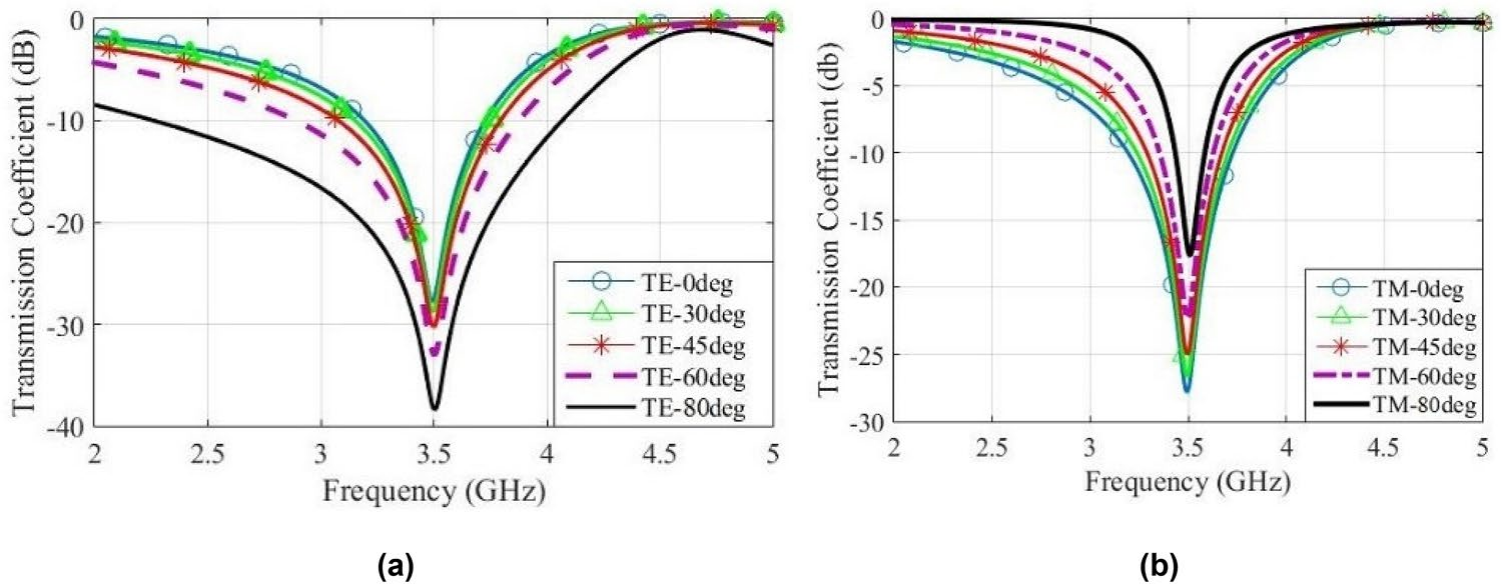
Transmission coefficients of the proposed FSS, (**a**) TE mode, (**b**) TM mode.

For the decrease of resonance frequency and maintenance of angular and polarization stabilities, the IDC may be used^21^. The proposed FSS unit cell is shown in Fig. 5, which is obtained by the superformula with the parameters *m* = 16, *n*_1_ = 0*.*75, *n* = 02, *n*_3_ = 30, *a* = 1 and *b* = 1. The dimensions of the unit cell are: enclosing square side *p* = 10 mm, width of metallic trace *w* = 0*.*2 mm, spacing of IDC traces *g* = 0*.*2 mm, width of petal *q*_1_ = 2*.*6 mm, *q*_2_ = 1*.*1 mm, spacing between two adjacent cells in the infinite FSS sample *s* = 0*.*2 mm and FR-4 substrate with dielectric constant *ε*_*r*_ = 4*.*3, height *h* = 1*.*6 mm and loss tangent tan *δ* = 0*.*02. Figure 6 shows the transmission coefficients of the proposed FSS for TE mode and TM mode. Observe that the proposed FSS unit cell has appropriate polarization and angular stability for the incident angles of 0° to 80° and also suitable frequency bandwidth (530 MHz at 0°).

## Analysis of the equivalent circuit of proposed FSS

**Figure 7:**
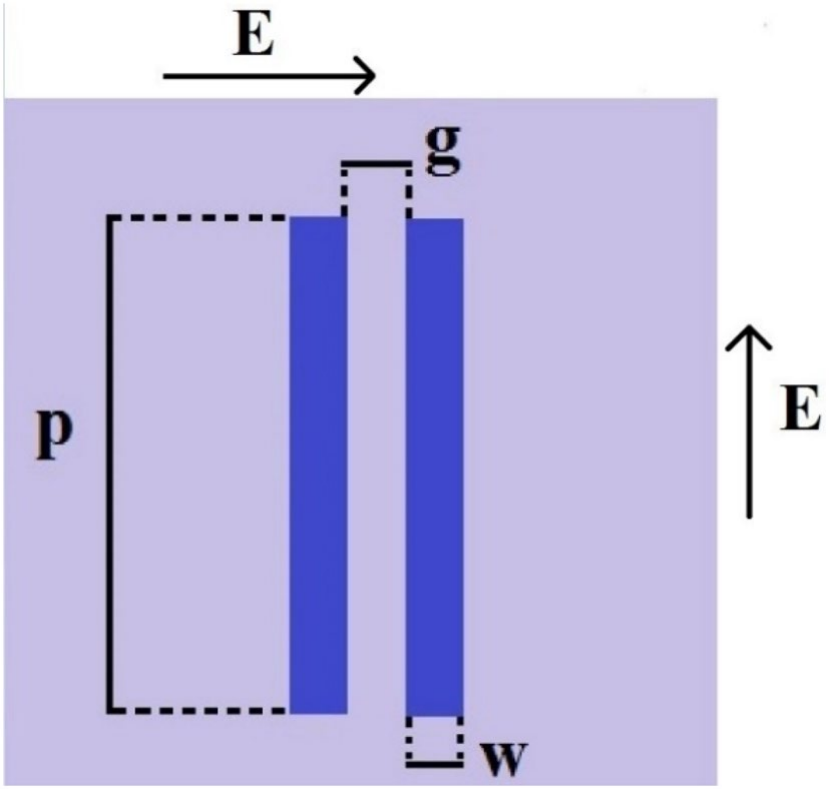
Parallel wires used as the inductor or capacitor based on the electric field direction.

To investigate the physical behavior of a unit-cell geometry and its dimensions on its performance together with a comparison with the full-wave simulation of its responses, an equivalent circuit of the proposed FSS is obtained. Indeed by the analysis and variation of components in the equivalent circuit, the desired transmission coefficient can be determined and their limitations identified, Then, by the variation of gaps between tracks and track widths and also with due consideration of fabrication limitations, the unit-cell maybe modeled. Indeed the equivalent circuit helps the designer to reduce the design efforts and simulation tasks for a particular structure with the desired specifications, The equivalent circuits actually provide some primary design guideline. The equivalent circuit configuration highly depends on the geometry of unit cell structure and its dimensions and also the polarization of incident wave^19^. However, in this section its equivalent circuit is presented for the polarization of TE (0°) 3.5 GHz. The values of equivalent circuit components are evaluated by the distributions of electric field and surface currents on the unit cell. When the incident electric field is perpendicular to the two parallel tracks, maximum electric field distribution and capacitance effect will appear in these regions. On the other hand, maximum current will be induced in a track parallel to the electric field and inductive effects will appear in the tracks carrying induced currents^4,22^. However, the capacitance between two adjacent unit cells depends on the polarization of incident angle, which will be considered in the equivalent circuit of FSS. Since the structure is periodic, half of the adjacent unit cells need be considered. The values of capacitive and inductive elements are due to the geometry of structure. Reference^22^ investigated the relation between capacitive and inductive elements with the geometry of structure and incident wave in strip line as shown in Fig. 7, where *L* is the strip inductance, *C* is the capacitance between the two adjacent patches, which is determined by the strip length *P*, the gap *g* between adjacent strips and the dielectric permittivity (*ε*), the strip width *w*, and the permeability *µ* of the structure.

2 $$C= {\varepsilon }_{0}{\varepsilon }_{r}\frac{2p}{\pi }cos\theta \left[lncosec\left(\frac{\pi g}{2p}\right)\right]$$

3 $$L= {\mu }_{0}{\mu }_{r}\frac{p}{2\pi }cos\theta \left[lncosec\left(\frac{\pi w}{2p}\right)\right]$$

In fact, the physical interpretation of proposed FSS behavior is provided by the help of equivalent circuit. It helps to interpret the variations of frequency responses of FSS as a function of geometrical configurations of FSS and also its polarization stability. Consequently, the equivalent circuit serves as an initial guide for the investigation of the frequency response of FSS.

**Figure 8:**
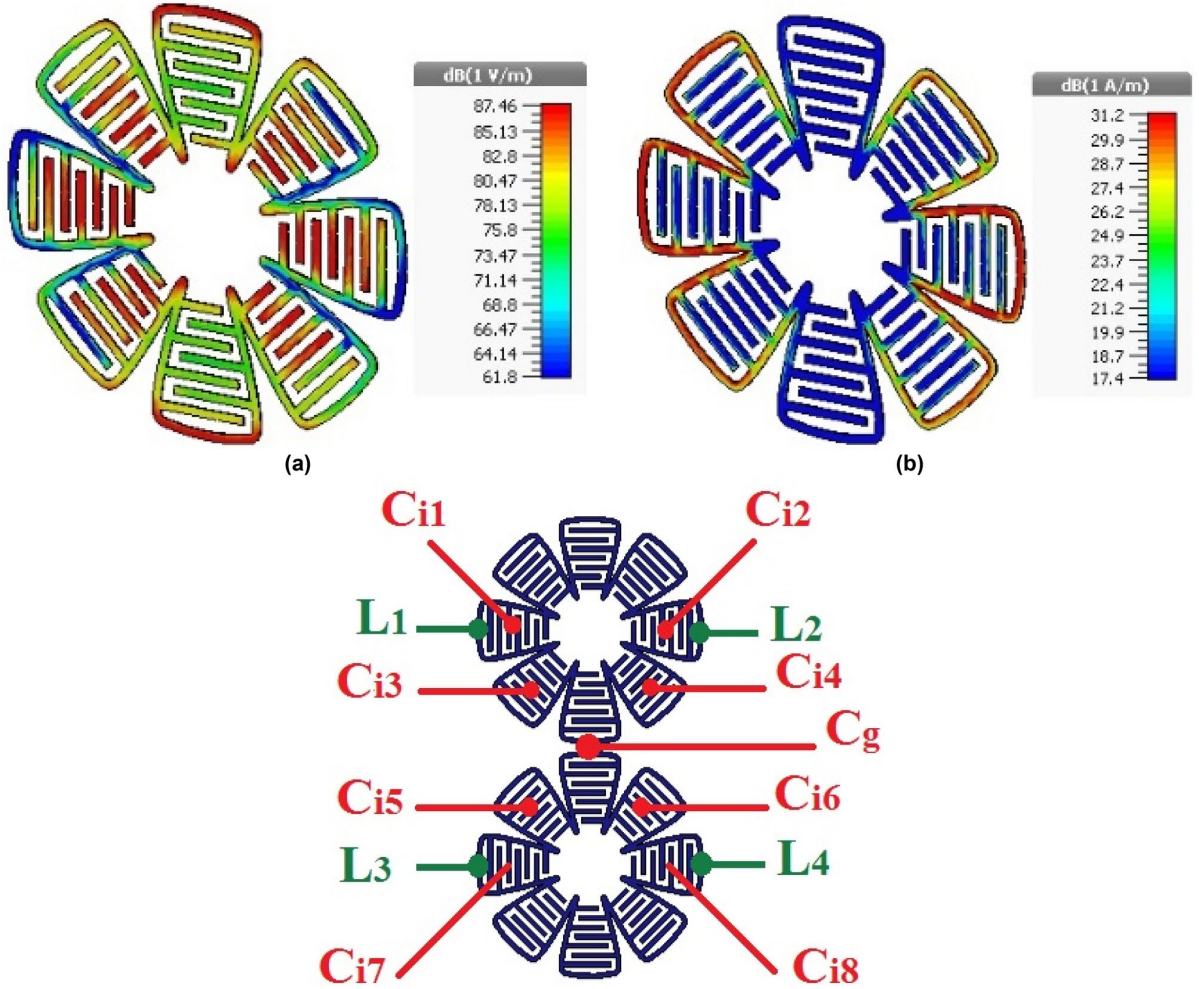
Simulation results of the proposed unit cell. (**a**) Electric field, (**b**) surface current, (**c**) capacitive and inductive elements.

**Figure 9:**
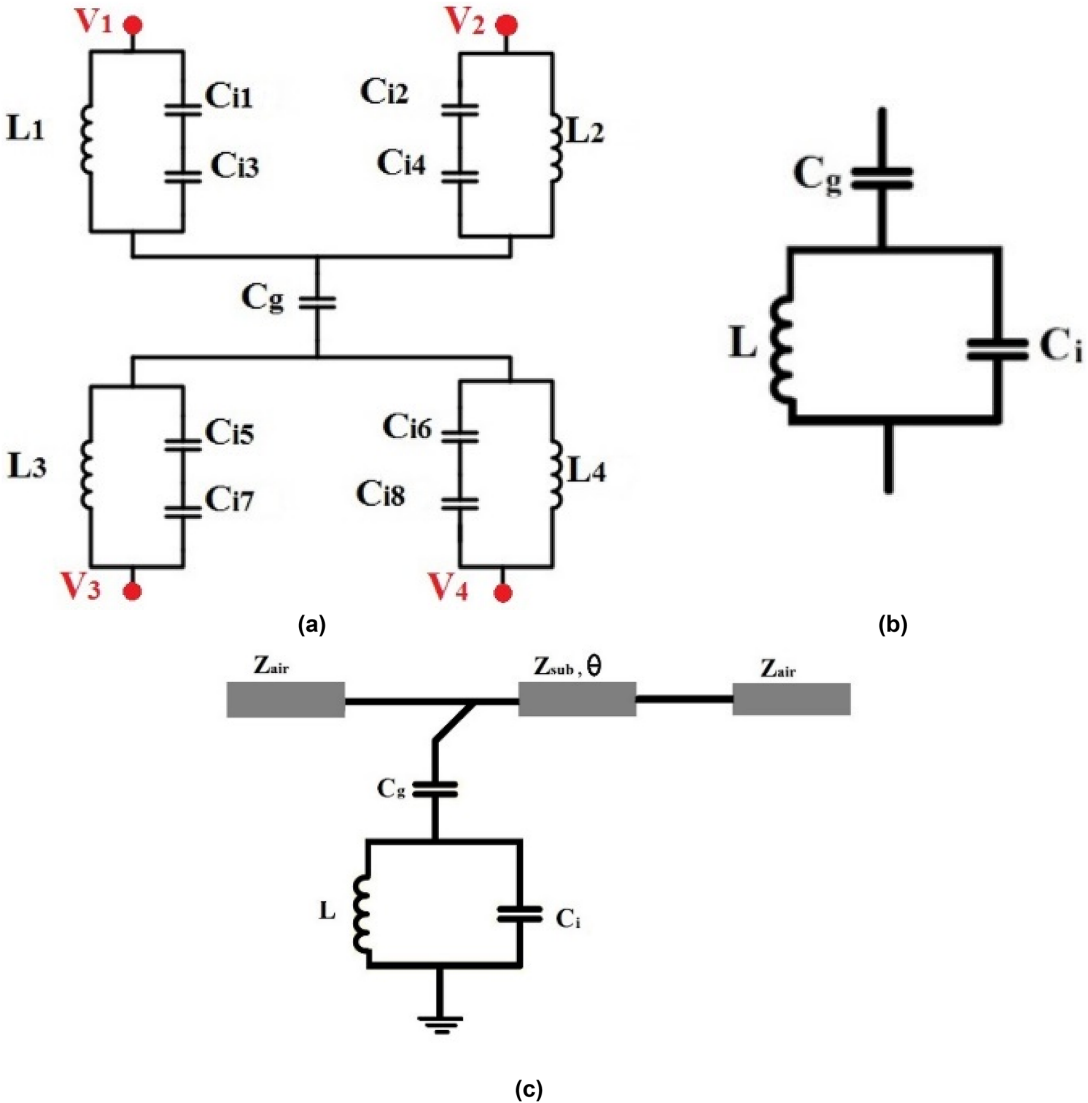
Equivalent circuit of proposed FSS. (**a**) The primary equivalent circuit of unit cell, (**b**) simplified form of equivalent circuit, (**c**) transmission line model.

Figure 8 shows the distributions of electric field (Fig. 8a) and surface currents (Fig. 8b) on the unit cell in the case of TE_0 deg at 3.5 GHz. Inductances and capacitances will appear on the unit cell according to the paths of currents and distribution electric field respectively. Due to the geometry of structure and current and electric field distribution on FSS, we have L_1_ = L_2_ = L_3_ = L_4_, C_i1_ = C_i2_ = C_i7_ = C_i8_ and C_i3_ = C_i4_ = C_i5_ = C_i6_ (Fig. 8c). Furthermore, the capacitors C_i1_, C_i2_, C_i5_ and C_i6_ are corrected in series with C_i3_, C_i4_, C_i7_ and C_i8_ respectively. Since the structure has geometrical symmetry and is periodic, equipotential points appear on it, then v_1_ = v_3_ and v_2_ = v_4_ as shown in Fig. 9. Observe that in a closed loop structure, the resonance frequency occurs when the circumference of the loop is equal to a multiple of a wavelength, ignoring the capacitive coupling of adjacent loops and IDC. Therefore, maximum and minimum absolute value of electric field occurs at $\lambda /4$ spacing. The electric field is minimum at points where v_1_ = v_3_ and v_2_ = v_4_ (Fig. 9a). These points are at the distance of $\lambda /2$ from each other, therefore they are equipotential. Consequently, the equivalent circuit simplifies to the circuit in Fig. 9b.

**Figure 10:**
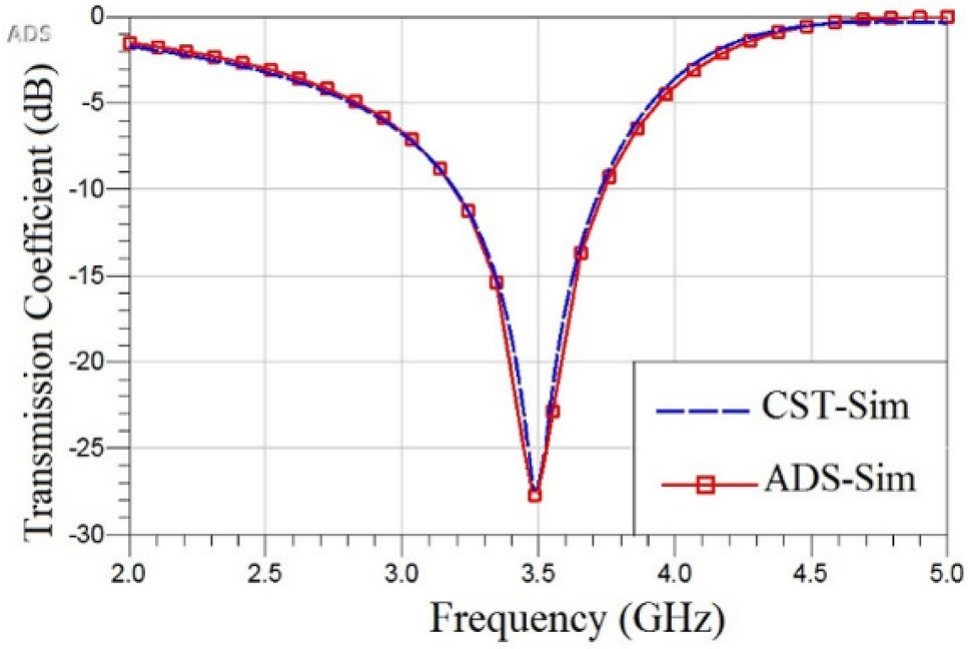
Comparison of transmission coefficients obtained by the full-wave simulation and equivalent circuit.

The values of elements of the equivalent circuit may be evaluated from the equivalent circuit in Fig. 9c. The free space on the two sides of unit cell may be modeled as a transmission line of characteristic impedance *Z*_*air*_ = 377 Ω and the characteristic impedance of substrate is *Z*_*sub*_=${Z}_{air}/\sqrt{{\varepsilon }_{r}}$ *≈* 180 Ω for FR-4. The type of substrate material and its height affect the value phase constant as $\theta =\beta l$. C_g_ is the capacitance between the unit cells, C_i_ is the capacitance between metallic interdigital tracks and L is the equivalent inductance of loop. Indeed the single-layer single-sided closed loop unit-cell has a simple equivalent circuit model (including series capacitors and inductors), but the model of complex structures will become more complicated. For the multiband unit-cells, the mutual effects of resonators should be considered together with the equivalent circuit of each resonator^19^. In the single-layer, double-sided layers and multilayer structures or those connected by vias, the effects of every side in the thin substrate or effect of vias should be considered in the equivalent circuits^4^. The proposed unit-cell is actually a closed loop shape with added capacitance (the capacitance and inductive elements are connected in parallel). Consequently, the equivalent circuit of this unit-cell is simpler than that in^23^. Figure 10 shows the results of the computer simulation by the full-wave computer simulation software CST^24^ and the transmission line model by the ADS simulation software^25^. These two results have excellent agreement. The values of elements of the equivalent circuit are given in Table 1. Observe that the value of *C*_*i*_ is larger than that of *C*_*g*_, because the electric field distribution in the IDC region is denser than that in the gap between unit cells.

**Table 1:** Values of elements of equivalent circuit.

*L*	*C*_*i*_	*C*_*g*_
2.35 nH	0.73 pF	0.15 pF

**Figure 11:**
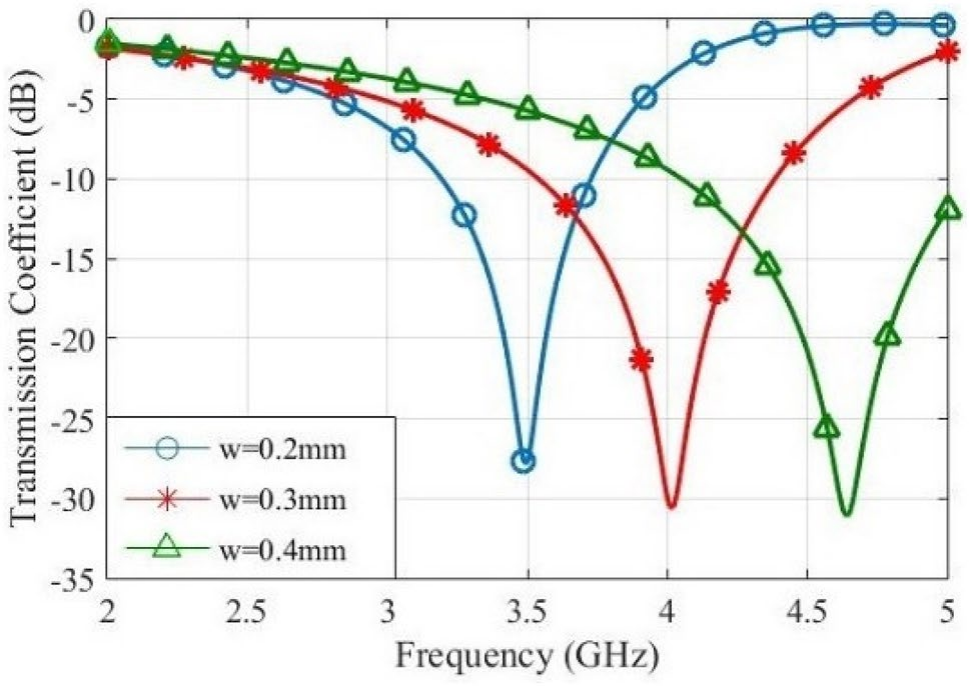
Effect of width of metallic traces on the transmission response.

A useful physical insight may be gained into the performance of unit cell, which could help for its design. The effect of variation of parameters *ε*_*r*_, *w*, *s* and *g* on the transmission coefficient of unit cell will be investigated by the devised equivalent circuit. Figure 11 shows the frequency response of transmission coefficient for various values of *w*. Observe that the increase of *w* leads to the increase of resonance frequency, since the increase of width of metallic tracks will decrease the equivalent inductance^22^. Furthermore, the number of IDCs will decrease leading to the decrease of *C*_*i*_. Also, the increase of inductance will decrease the bandwidth.

**Figure 12:**
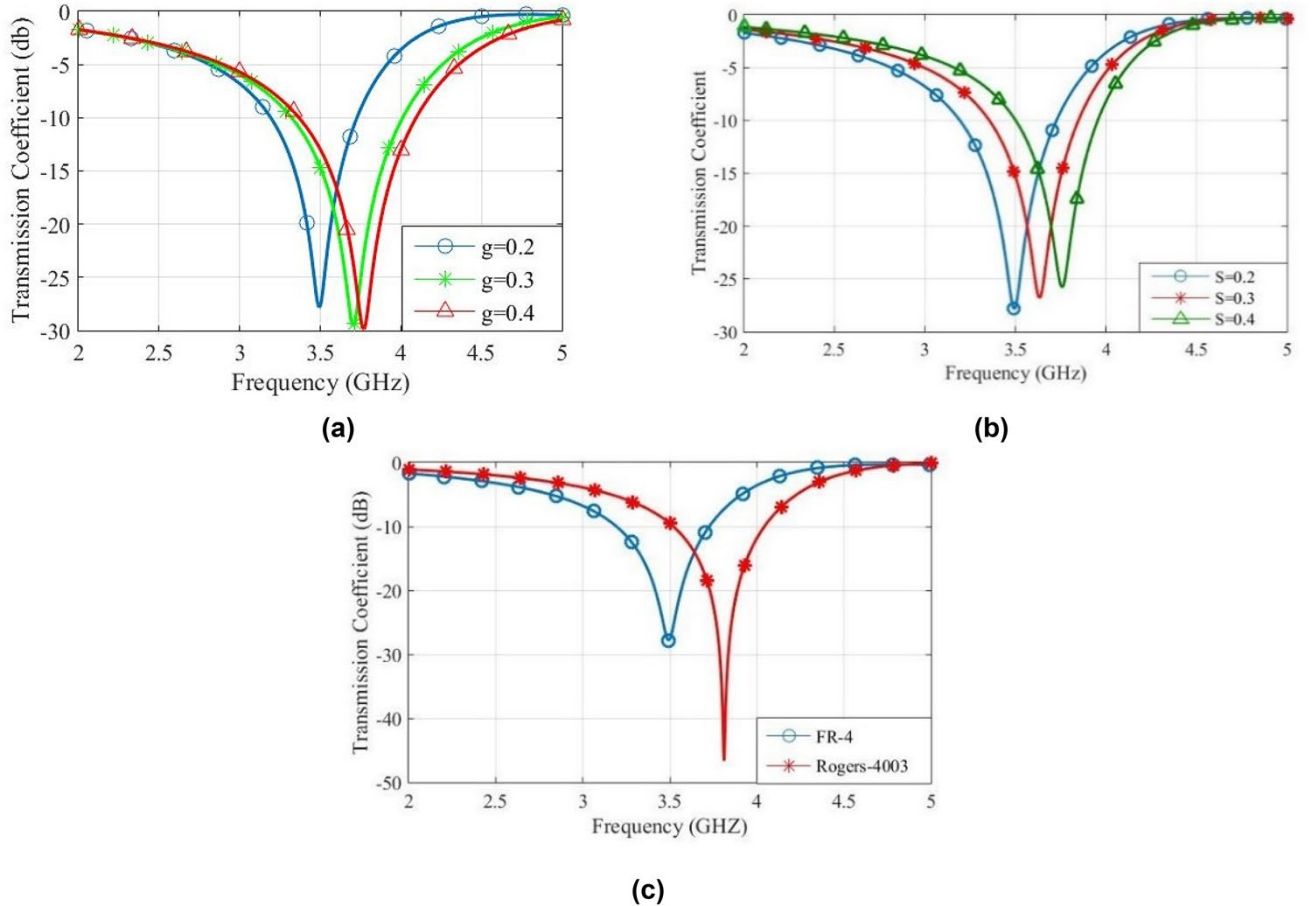
Transmission coefficient for different values of, (**a**) *g*, (**b**) *s*, and (**c**) *ε*_*r*_.

The parameters *ε*_*r*_, *s* and *g* affect capacitances of the equivalent circuit, due to the formula *C* = *ε*_*r*_*ε*_0_*A*/*d*^22^. Therefore, the increase of capacitance will decrease the resonance frequency. Figure 12 shows the effect of values of *ε*_*r*_, *s* and *g* on the resonance frequency of FSS unit cell. Observe that the increase of interdigital capacitance decreases the bandwidth and increases the slope of roll-off. Also, the increase of capacitance between the adjacent unit cells will lead to the increase of bandwidth and the decrease of slope of roll-off. The variations of *ε*_*r*_ affect the effective dielectric constant *ε*_*eff*_ of capacitances. Also, the equivalent impedance of substrate affects the transmission line. Observe that the increase of *ε*_*r*_ decreases the slope of roll-off.

Table 2 is presented to highlight the advantages of the proposed unit-cell compared to those in the available literatures. Observe in Table 2 that the proposed unit cell has a better angular-stability compared to those reported in the literature. Recently, high angular-stability FSSs have been fabricated based on 2.5-dimensional structures. Note that, for a 2.5-dimensional FSS, the angular stability is due to its miniaturization, which is obtained by exploiting via-hole structures connecting tracks in two sides of the dielectric host medium. Using 2.5-dimensional structures may complicate the fabrication process. However, the proposed natural geometry for FSS in this paper is fully investigated for the achievement of its characteristic behavior and angular stability.

**Table 2:** Performance of the proposed FSS compared to the reported literature.

References	Unit cell size	Structure	− 10 dB fractional BW	Angular stability	Polarization stability
^26^	0.076 λ_0_	Multilayer	10%	60°	Yes
^27^	0.33λ_0_	Single layer Both side	106%	60°	Yes
^28^	0.1 λ_0_	Single layer Single side	18.7%	45°	Yes
^29^	0.084 λ_0_	Single layer single side	Not reported	45°	Yes
^30^	0.63 λ_0_	2.5-dimansional	82.4%	45°	Yes
^31^	0.027 λ_0_	2.5-dimansional	17.8%	75°	Yes
^32^	0.27 λ_0_	Single layer single side	40%	60°	No
Proposed FSS	0.11 λ_0_	Single layer single side	15%	80°	Yes

## Fabrication and measurement

For the proof of concept, the proposed FSS is designed and it prototype model is fabricated and it is measured in anechoic chamber. The dimensions of the fabricated prototype model is 350 mm × 350 mm include 35 × 35 unit cell. The measurement frequency range is 3–4 GH that equal to 7.5–10 cm for free space wavelength. In case of usage of FR-4 substrate with ${\varepsilon }_{r}=4.3$ and loss tangent = 0.02, the effective wavelength is equal to 4.57–6.1 cm respectively due to $\lambda = \left({\lambda }_{0}\left(\sqrt{\left(1+{\varepsilon }_{r}\right)/2}\right)\right)$. Note that the dimension of fabricate FSS is about six times longer than longest wavelength of measurement range. The same antennas with 54-degree 3-dB bandwidth and less than 1 m far field range in 3–4 GHz is used for transmit and receive antennas. To achieve the best measurement precision, the periphery of the fabricated FSS under test is covered by absorber material to block and dissipate any stray waves, in order to direct the wave solely through the FSS window area. Such a procedure is repeated for each angle of incidence. For measurement at any angle, the transmit and receive antennas are fixed and the FSS plate is rotated. In all measurements, the FSS plate is surrounded by absorber screens so that the path of waves are completely blocked and absorbed in its neighborhood. By this method, the direct path between the transmitter and receiver is completely blocked by the FSS resulting in better measurement precision.

For a simple and accurate measurement procedure, we selected the test set-up consisting of source and spectrum devices. For the calibration, first the transmission coefficient for maximum reception between the transmit and receive antennas is measured from 3 to 4 GHz. It is set for the reference value of transmission coefficient of simulation for TE (0°). Then the fabricated FSS is placed in the midpoint using absorber screens around the system between the transmit and receive antennas and again the transmission coefficient is measured. The frequency spectrum plot gives the transmission coefficient of FSS structure for TE (0°). The procedure is repeated for other incident angles with the same reference. The measured FSS transmission coefficients agree well with the computer simulation values. For valid measurement results, the FSS structure should be positioned in the far field regions of the transmitter and receiver antennas.

**Figure 13:**
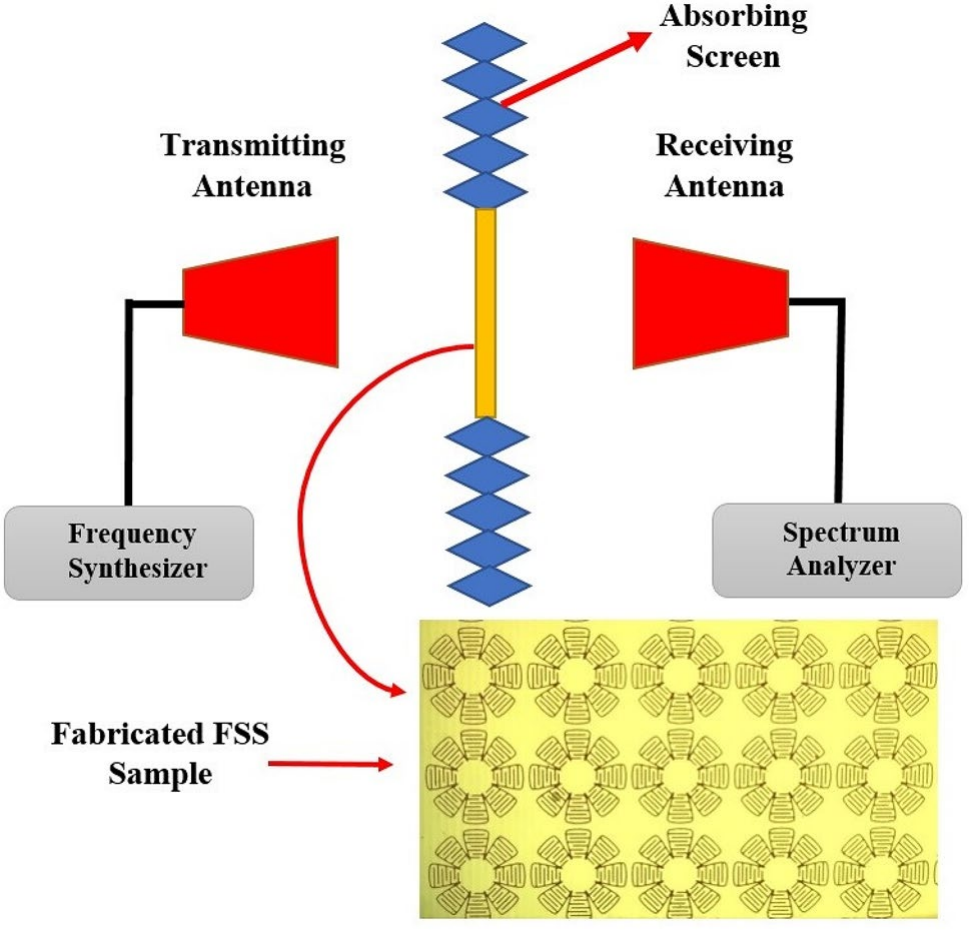
Fabricated prototype and test set-up model.

Unlike the Network Analyzer that uses a calibration kit for measurements, the Spectrum Analyzer doesn’t use any calibration kit. Indeed the calibration is merely used in measurement devices to eliminate losses of cables connecting the device-under-test and also produce and set specified phases in some measurements, which require the realization of some phase behavior^32,33^. In our specific measurements, the transmission coefficient diagram is our main goal, but the phase diagram is not desired. The measurement of cable losses and applying them to the responses obtained by the spectrum analyzer, provides the actual transmission coefficient response, which is identical to that of the calibrated Network Analyzer. On the other hand, the frequency of test setup is not high and the insertion losses are not noticeable. Consequently, the measurements by the Network Analyzer and Spectrum Analyzer give identical responses. The free space loss in both methods and set-ups is the same. The measurement of free space loss between the TX and RX antennas in the absence of FSS screen is first conducted. Then, the maximum power received from the transmitter with the FSS located in its place is equal to the actual simulation values in the frequency band under test. Figure 13 shows the fabricated prototype and the test set-up system.

**Figure 14:**
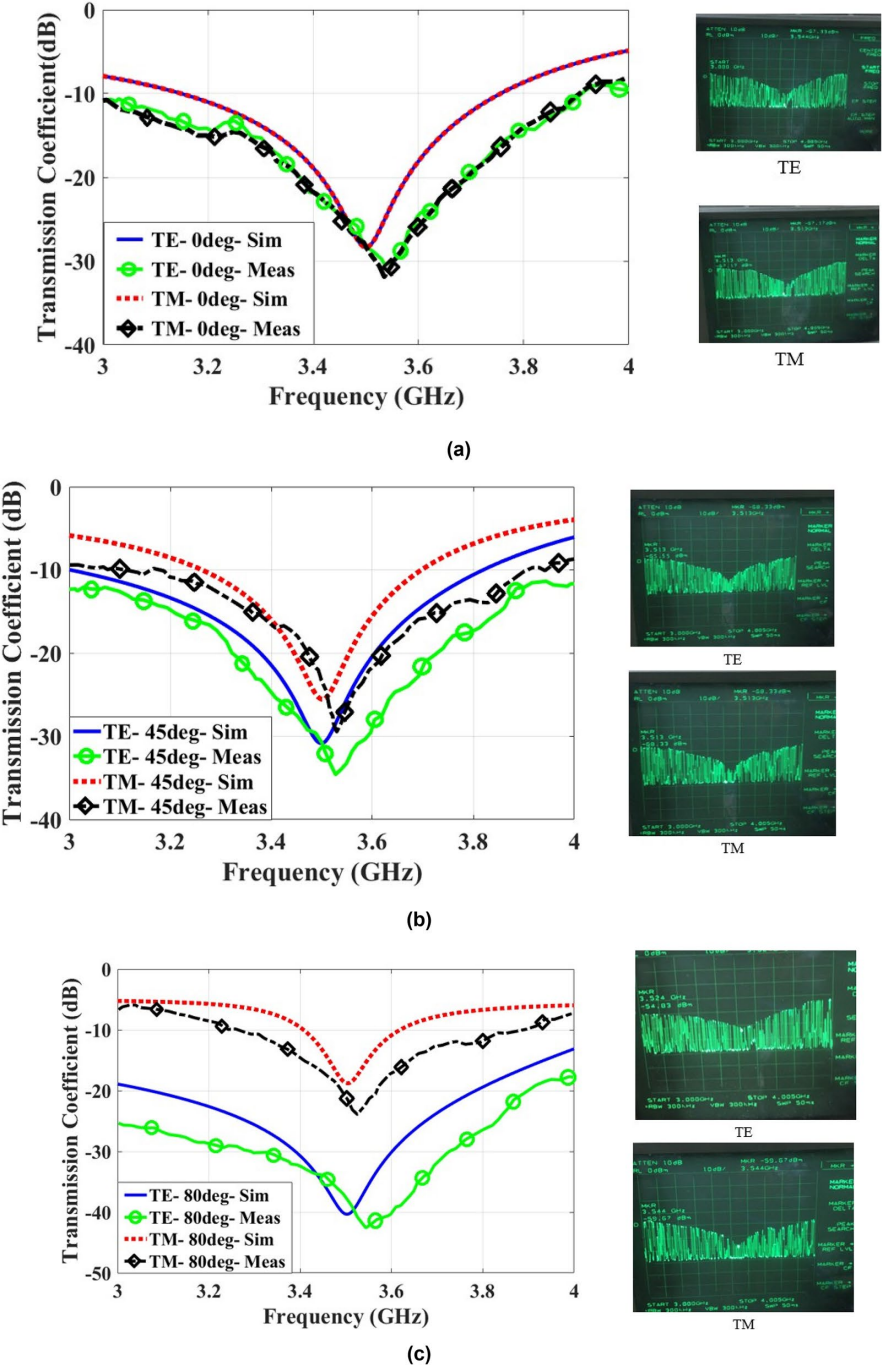
Measurement data for TE and TM modes, (**a**) 0°, (**b**) 45°, and (**c**) 80°.

Figure 14 shows the measurement data for TE and TM models for the incidence angles 0°, 45° and 80°. Observe that the resonance frequency is 3.52 GHz. Any deviation from the design specifications may be due to the imprecision of fabrication and inaccuracy of measurements. However, the measurement data agree well with the simulation results and shows the angular stability of the proposed FSS and the independence of response of unit cell to incident angle, although the resonant frequency shifts.

## Conclusion

The use of superformula is proposed to devise structures for the frequency selective surface (FSS). As an example, the geometry of FSS is designed in the shape of petals incorporating interdigital capacitances, which provide excellent miniaturization and also angular and polarization stabilities of incident waves. An equivalent circuit is devised for the proposed FSS unit cell for the investigation of distributions of electric field and surface currents on it. The values of its capacitors and inductors are determined. The effect of variations of the geometry of FSS on the frequency responses are investigated. Consequently, the bandwidth and roll-off of the frequency responses of the proposed FSS unit cell may be adjusted by the variation of its dimensions. A prototype model of the proposed FSS is fabricated and measured as the proof of concept. It is shown that the prototype possesses both angular and polarization stabilities. Its performances are evaluated by the equivalent circuit, full-wave simulation and actual measurement, which agree well in the specified frequency bandwidth.

## References

[1] Munk BA (2000). Frequency Selective Surface Theory and Design.

[2] Ghosh S, Srivastava KV (2018). Broadband polarization-insensitive tunable frequency selective surface for wideband shielding. IEEE Trans. Electromagn. Compat..

[3] Zhao Z, Shi H, Guo J, Li W, Zhang A (2017). Stopband frequency selective surface with ultra-large angle of incidence. IEEE Antennas Wirel. Propag. Lett..

[4] Khajevandi S, Oraizi H, Amini A, Poordaraee M (2019). Design of miniaturised-element FSS based on 2.5-dimensional closed-loop hilbert fractal. IET MAP.

[5] Vásquez-Peralvo JA, Fernández-González J-M, Valtr P, Rigelsford JM (2020). Inductive frequency selective surface: An application for dichroic sub-reflectors. IEEE Access.

[6] Zhang Y (2019). A broadband tunable frequency selective surface absorber for oblique incidence applications. J. Phys. D Appl. Phys..

[7] Liu T, Kim S-S (2019). Ultrawide bandwidth electromagnetic wave absorbers using a high-capacitive folded spiral frequency selective surface in a multilayer structure. Sci. Rep..

[8] Liu N, Sheng X, Zhang C, Guo D (2018). Design of frequency selective surface structure with high angular stability for radome application. IEEE Antennas Wirel. Propag. Lett..

[9] Yin W, Zhang H, Zhong T, Min X (2018). A novel compact dual-band frequency selective surface for gsm shielding by utilizing a 2.5-dimensional structure. IEEE Trans. Electromagn. Compat..

[10] Gao B, Yuen MMF, Ye TT (2017). Flexible frequency selective metamaterials for microwave applications. Sci. Rep..

[11] Hashemi S, Abdolali A (2017). Room shielding with frequency-selective surfaces for electromagnetic health application. Int. J. Microw. Wirel. Technol..

[12] Li T, Li D, Zhou L, Li E (2018). Miniaturised FSS structure with excellent angular stability based on strong coupling for millimetre-wave communication. Electron. Lett..

[13] Brito, D. B., Araújo, L. M., D’Assunção, A. G. & Maniçoba, R. H. C. A minkowski fractal frequency selective surface with high angular stability. In *2013 SBMO/IEEE MTT-S International Microwave Optoelectronics Conference (IMOC)* 1–4. 10.1109/IMOC.2013.6646577 (2013).

[14] Anwar RS, Wei Y, Mao L, Ning H (2019). Miniaturised frequency selective surface based on fractal arrays with square slots for enhanced bandwidth. IET Microwaves Antennas Propag..

[15] Gielis J (2003). A generic geometric transformation that unifies a wide range of natural and abstract shapes. Am. J. Bot..

[16] Poordaraee, M., Oraizi, H., Khajevandi, S. & Glazunov, A. A. Systematic design of a circularly polarized microstrip antenna using a shape super-formula and the characteristic mode theory. In *2018 18th Mediterranean Microwave Symposium (MMS)* 47–50. 10.1109/MMS.2018.8612068 (2018).

[17] Poordaraee, M., Oraizi, H., Khajevandi, S. & Hodjat-Kashani, F. Systematic octopus-shape antenna design with circular polarization by characteristic mode theory. In *12th European Conference on Antennas and Propagation (EuCAP 2018)* 1–5. 10.1049/cp.2018.1016 (2018).

[18] Whiting EB, Campbell SD, Mackertich-Sengerdy G, Werner DH (2021). Dielectric resonator antenna geometry-dependent performance tradeoffs. IEEE Open J. Antennas Propag..

[19] Khajevandi S, Oraizi H, Poordaraee M (2018). Design of planar dual-bandstop FSS using square-loop-enclosing superformula curves. IEEE Antennas Wirel. Propag. Lett..

[20] Kesavan A, Mantash M, Denidni TA (2019). Supershaped reconfigurable frequency-selective surfaces using cantilever enable switches. Microw. Opt. Technol. Lett..

[21] Ghosh S, Lim S (2019). A miniaturized bandpass frequency selective surface exploiting three-dimensional printing technique. IEEE Antennas Wirel. Propag. Lett..

[22] Marcuvitz N (1951). Waveguide Handbook.

[23] Khajevandi A, Oraizi H (2021). Design of frequency selective surface based on minkowski fractal and interdigital capacitance. Electron. Lett..

[24] Computer simulation technology (cst). http://www.cst.com.

[25] Advanced design system (ads). http://www.keysight.com.

[26] Hussein M, Zhou J, Huang Y, Al-Juboori B (2017). A low-profile miniaturized second-order bandpass frequency selective surface. IEEE Antennas Wirel. Propag. Lett..

[27] Sood D, Tripathi CC (2018). Polarization insensitive compact wide stop-band frequency selective surface. J. Microw. Optoelectron. Electromagn. Appl..

[28] Farooq U, Iftikhar A, Shafique MF, Mughal MJ (2019). A miniaturized and polarization insensitive FSS and CFSS for dual band WLAN applications. AEU Int. J. Electron. Commun..

[29] Ali H, Shafique MF, Riaz L, Khan MA (2018). A compact, stable, and angular independent dual-band frequency-selective surface for WIFI applications. Microw. Opt. Technol. Lett..

[30] Hussain T, Cao Q, Kayani JK, Majid I (2017). Miniaturization of frequency selective surfaces using 2.5-D knitted structures: Design and synthesis. IEEE Trans. Antennas Propag..

[31] Bakir M, Delihacioglu K, Karaaslan M, Dincer F, Sabah C (2016). U-shaped frequency selective surfaces for single- and dual-band applications together with absorber and sensor configurations. IET Microwaves Antennas Propag..

[32] Yuan Y, Sun S, Zhang K, Ding X, Ratni B, Wu Q, Burokar S, Qui C (2020). A Fully phase-modulated metasurface as an energy-controllable circular polarization router. Adv. Sci..

[33] Zhang K, Wang Y, Burokur S, Wu Q (2021). Generating dual-polarized vortex beam by detour phase: From phase gradient metasurfaces to metagratings. IEEE Trans. Microwave Theory Techn..

